# Exploring the evolution of evidence synthesis: a bibliometric analysis of umbrella reviews in medicine

**DOI:** 10.1097/MS9.0000000000003034

**Published:** 2025-02-26

**Authors:** Sandeep Samethadka Nayak, Ehsan Amini-Salehi, Michael T. Ulrich, Yasmin Sahli, Micah Fleischman, Masum Patel, Mahdi Naeiji, Hasan Maghsoodifar, Seyed Amir Hossein Sadeghi Douki, Abdolhadi Alotaibi, Niloofar Faraji, Soheil Hassanipour, Mohammad Hashemi, Mohammad-Hossein Keivanlou

**Affiliations:** a Division of Hospital Medicine, Department of Internal Medicine Yale New Haven Health, Bridgeport, CT, USA; b Gastrointestinal and Liver Diseases Research Center, Guilan University of Medical Sciences, Rasht, Iran; c Department of Medicine, Riverside University Health System Medical Center, Moreno Valley, CA, USA; d Yale New Haven Health, Bridgeport Hospital, Bridgeport, CT, USA; e Department of Physiology and Neurobiology, University of Connecticut, Storrs, CT, USA; f Byramjee Jeejeebhoy Medical College 34, Jalaramnagar Society, Mehsana, Gujarat, India; g Student Research Committee, School of Medicine, Shahroud University of Medical Sciences, Shahroud, Iran; h Department of Medicine and Surgery, Vision Colleges, Riyadh, Saudi Arabia; i Cardiovascular Research Center, Hormozgan University of Medical Sciences, Bandar Abbas, Iran

**Keywords:** bibliometric analysis, evidence synthesis, meta-analysis, research trends, scientific publication pattern, systematic reviews, umbrella reviews

## Abstract

**Background:**

Umbrella review studies have become increasingly vital in evidence synthesis, offering a comprehensive overview by analyzing multiple systematic reviews and meta-analyses. This bibliometric study aimed to delineate the growth and thematic evolution of umbrella reviews within evidence-based medicine, illuminating their integral role in synthesizing high-level evidence.

**Methods:**

Utilizing the Web of Science Core Collection, we performed a search for publications on umbrella reviews, identifying relevant articles through a refined strategy. Analytical tools including VOS Viewer and CiteSpace were employed to visualize connections and trends among the gathered data, converting intricate bibliometric information into comprehensible visual maps.

**Results:**

Our search yielded 2965 pertinent publications, highlighting a marked growth in research output, particularly from 2010 to 2023. The United States, United Kingdom, and China were predominant in this field, with leading institutions like King’s College London and the University of Toronto at the forefront. The analysis identified major journals such as *BMJ Open* and *PLOS One* as key publishers. Co-citation and keyword analysis revealed current research focuses, with recent trends emphasizing COVID-19 and mental health. The study also uncovered a robust international collaboration network, underscoring the global impact of umbrella reviews.

**Conclusion:**

This bibliometric analysis confirms the expanding influence and utility of umbrella reviews in medical research and decision-making. By charting the evolution and current trends in this field, our study not only showcases the geographical and institutional distribution of research but also guides future scholarly efforts to advance evidence synthesis methodologies.

## Introduction

In evidence-based medicine and scientific research, traditional systematic reviews and meta-analyses have been essential for summarizing and integrating research findings^[1–3]^. However, in recent years, a novel approach known as umbrella reviews has emerged, offering a more comprehensive perspective on evidence synthesis^[4–8]^. Umbrella reviews, also termed overviews of reviews, systematically gather and analyze evidence from multiple systematic reviews or meta-analyses within a specific field or topic area, providing a comprehensive understanding of the available evidence^[9–15]^. Thus, umbrella reviews prevail as one of the highest levels of evidence synthesis utilized at present^[16–19]^. The rise of umbrella reviews reflects the need to manage the growing volume of scientific literature and overlapping systematic reviews. By synthesizing findings from multiple meta-analyses, they offer a comprehensive overview of evidence within a specific field^[20–23]^.

**Highlights**

- This study unveiled a significant surge in the publication of umbrella reviews from 2010 to 2023, emphasizing their critical role in synthesizing evidence for medical decision-making.
- This study revealed the predominant contribution of institutions and researchers from the United States, United Kingdom, and China, showcasing the global scale and collaborative nature of research in evidence synthesis.
- This study identified emerging research trends and the growing importance of umbrella reviews in addressing complex medical topics, such as COVID-19 and mental health, through advanced bibliometric methods.

Bibliometric analyses are powerful tools for evaluating research trends and providing insights into the structure, dynamics, and progression of scientific inquiry^[24]^. Bibliometric analyses can identify the impact, trends, and gaps in the body of knowledge by methodically examining publishing patterns, citation networks, and topic evolutions within the scientific literature. A deeper understanding of the scientific world surrounding certain research fields is made possible by this methodological approach, which enables an objective evaluation of research activity and influence^[25–27]^.

Conducting bibliographic studies on umbrella reviews is crucial for several reasons. It systematically identifies and evaluates existing literature, offering insights into the depth of research in evidence synthesis. These studies also reveal trends and patterns in the application of umbrella reviews across disciplines, highlighting their evolution and relevance. Additionally, bibliographic analyses uncover gaps, methodological limitations, and inconsistencies, guiding future research to improve the quality and rigor of evidence synthesis^[28]^. By analyzing the trends, geographical and institutional distribution, and research focuses within the field of umbrella reviews, we seek to better clarify their role and significance in medical research. Our study aimed to investigate the understanding of umbrella reviews’ influence in the domains of evidence-based practice and decision-making through a bibliometric study.

## Methods

### Data collection process

**Figure 1.**
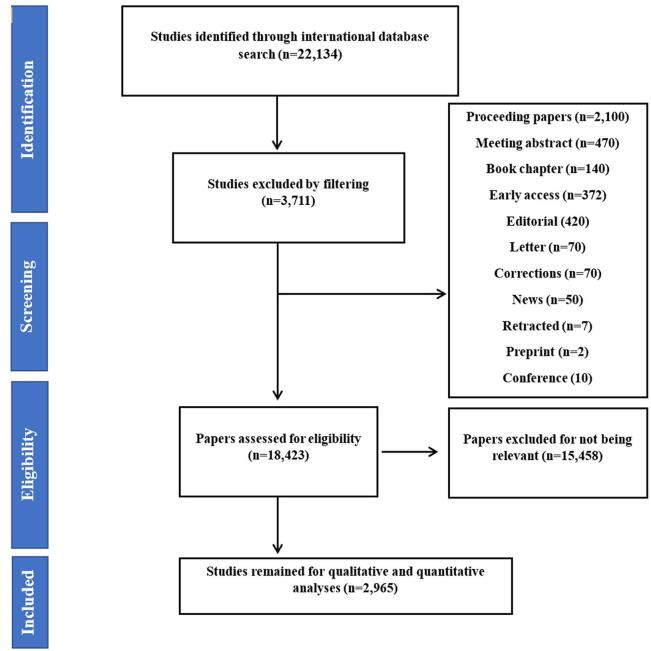
Study selection process.

On 7 March 2024, we searched for the Web of Science Core Collection as our primary database to perform our research on the published umbrella reviews. One notable feature of this database was its comprehensive data collection, which included over 12 000 credible journals^[29–31]^. As shown in Supplemental Digital Content Table S1, available at: http://links.lww.com/MS9/A735, we devised a complex search strategy that used the following terms: “Umbrella review,” “Overview of systematic reviews,” “Overview of meta-analyses,” “Overview of reviews,” “Meta-analysis in meta-analyses,” “Meta-umbrella,” and “Umbrella” to optimize the effectiveness of the search. The selected studies had to meet two criteria: being an umbrella review and addressing a medical topic, of which, 22 134 items were found. We next narrowed our selection to 2965 relevant papers by eliminating conference papers, letters, editorials, book chapters, pre-publication papers, and studies unrelated to our aim (Fig. 1).

**Figure 2.**
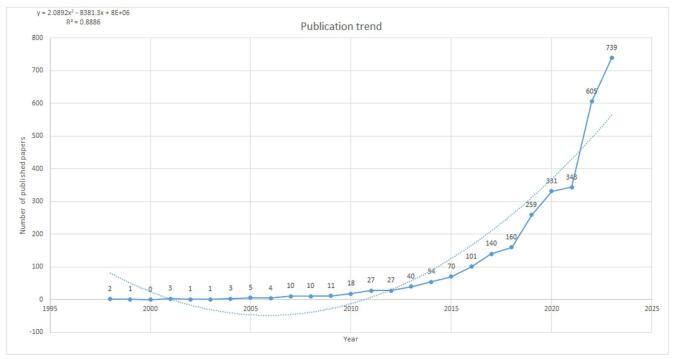
Histogram displaying yearly production output (continuous line) and a time trend curve (polynomial trend line, dotted line) illustrating this output.

### Data analysis

Using VOS viewer and CiteSpace, all pertinent documents obtained from the Web of Science Core Collection were transformed to Microsoft Excel 2019 and plain text formats for analysis. The Center for Science and Technology Studies at Leiden University in the Netherlands produced VOS viewer, which is a highly effective tool for scientometric network analysis. This software is outstanding at producing maps based on network data and visual insights, which makes it easier to comprehend the connections between academic literature. It is skilled at creating network diagrams that connect many kinds of academic entities, including co-citation, co-occurrence, citation, and bibliographic coupling, among publications, journals, authors, research institutes, nations, and keywords.

The Drexel University software CiteSpace, employed the Java programming language to provide citation visualization analysis based on scientometrics and data visualization principles. CiteSpace predominantly utilized bibliographic data from sources like Web of Science and PubMed. The data underwent preprocessing, which included session identification and analysis of sessions aggregated by IP. To analyze trends over time, CiteSpace divided the dataset into distinct time slices. Users can set thresholds for citation counts, co-citation counts, and co-citation coefficients to refine the data. CiteSpace created various types of networks, such as co-citation networks and heterogeneous networks that include terms and articles. Visualization methods included cluster views for co-citation networks, time-zone views for temporal pattern analysis, and citation tree-ring visualizations for clear and interpretable representations of citation trends^[32–35]^. CiteSpace helped individuals comprehend how scientific research was changing by giving citation networks a visual representation. This helped to identify important trends, important studies, and new topics within the massive body of academic communication^[34]^.

## Results

### Publication output and temporal trend

Fig. 2 illustrates the annual publication trends and temporal evolution of research on umbrella reviews since 1998. From 1998 to 2010, the field experienced steady growth, followed by a marked acceleration between 2010 and 2020. Notably, since 2016, annual publications have consistently surpassed 100, peaking in 2023 with over 700 papers, an unprecedented surge in scholarly output. This pattern highlighted the growing interest in Umbrella reviews, which entered a phase of rapid advancement due to their research significance and potential for further exploration.

### Analysis of journals

Our findings revealed that a total of 1180 journals contributed to the publication of umbrella reviews. Among these, 37 journals demonstrated significant engagement, each publishing more than 10 articles. Notably, *BMJ Open* led the field with an impressive total of over 100 umbrella reviews, followed closely by other prominent journals such as *PLOS One* and *Systematic Reviews*.

**Figure 3.**
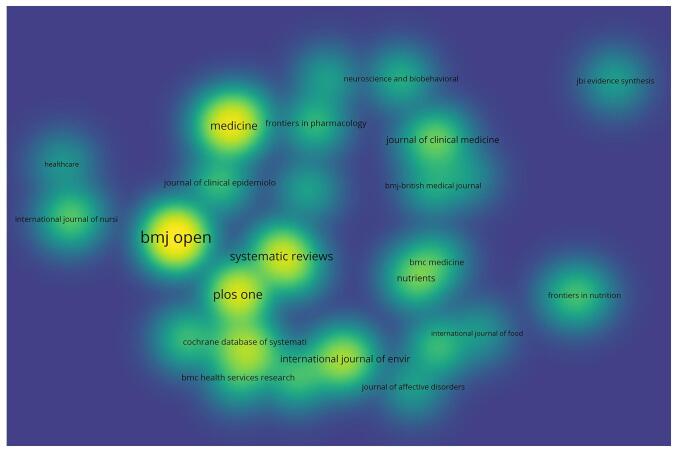
The density plot of top journals with publishing more than 10 umbrella reviews.

**Figure 4.**
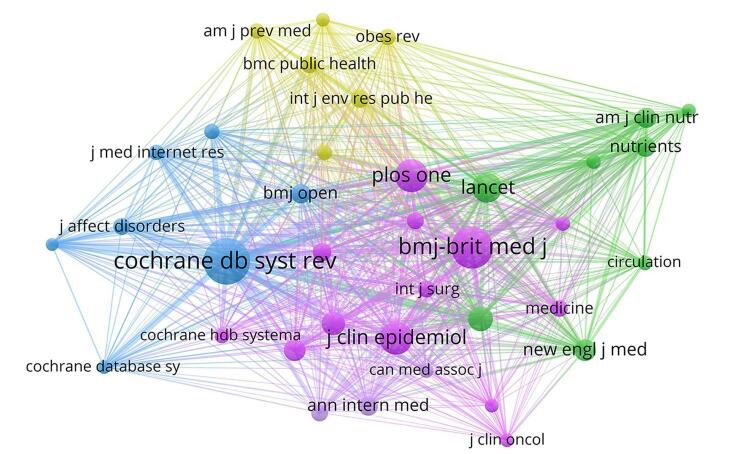
The network visualization of co-cited journals in the field of umbrella reviews.

Co-citation analysis is a useful technique for revealing the relationships between academic papers; it shows how the frequency of co-citations in a journal can indicate its impact on a particular field of study. With an emphasis on the relationships between publications, this method sheds light on the scholarly significance and topical applicability of journals in the field. We found a total of 24 951 co-cited journals. Out of these, the *Cochrane Database of Systematic Reviews* was the leading journal, followed by the *BMJ-British Medical Journal* and *Journal of Clinical Epidemiology*. Supplemental Digital Content Table S2, available at: http://links.lww.com/MS9/A735 provides a detailed overview of the top 10 leading journals and co-cited journals that have been actively contributing to umbrella reviews. Moreover, Fig. 3 offers a visual representation of journals that have published more than 10 umbrella reviews and Fig. 4 presents the network visualization of co-cited journals.

**Figure 5.**
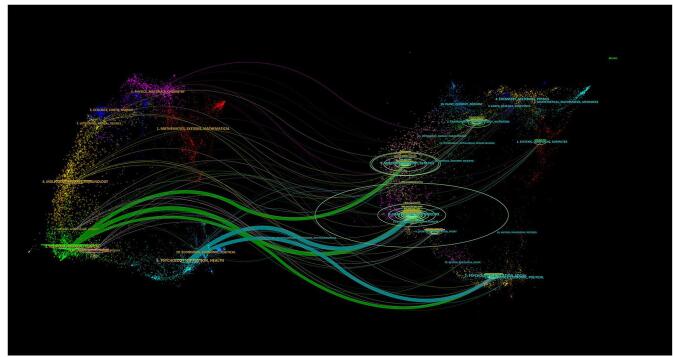
The dual-map overlay of journals in the field of umbrella reviews.

The dual-map overlay, demonstrating the interrelations among journals, showcased the citation dynamics. Journals citing others were positioned on the left side, while those being cited were on the right. As depicted in Fig. 5, the visualization unveils five main citation pathways, denoted by three green and two blue trajectories. The dual-map overlay indicated that research articles from molecular/biology/genetics journals received substantial citations from publications in the medicine/medical/clinical sector. Moreover, articles published in health/nursing/medicine journals were frequently cited by both medicine/medical/clinical and psychology/education/health journals. Additionally, articles from psychology/education/social journals were commonly cited by both medicine/medical/clinical and psychology/education/health journals. This mapping highlighted the interdisciplinary character of scientific research and its dissemination by highlighting the relationships between and effects of several domains on one another.

### Analysis of countries, regions, and institutions

The analysis identified a total of 112 countries that have contributed to umbrella reviews. Among these countries, 52 have contributed to more than 10 papers. England had the highest number of papers, followed by the USA, and China. There was a strong collaboration between England and the USA, England and Australia, England and Italy, the USA and Canada, the USA and Australia, Canada and England, and China and the USA. Table 1 displays the top 10 countries in umbrella reviews along with the number of their publications.

**Table 1:** Top 10 leading countries in the field of umbrella reviews

Number	Country	Number of publications
1	England	648
2	USA	557
3	China	490
4	Australia	364
5	Canada	339
6	Italy	321
7	Spain	239
8	Germany	230
9	Netherland	172
10	Brazil	160

**Figure 6.**
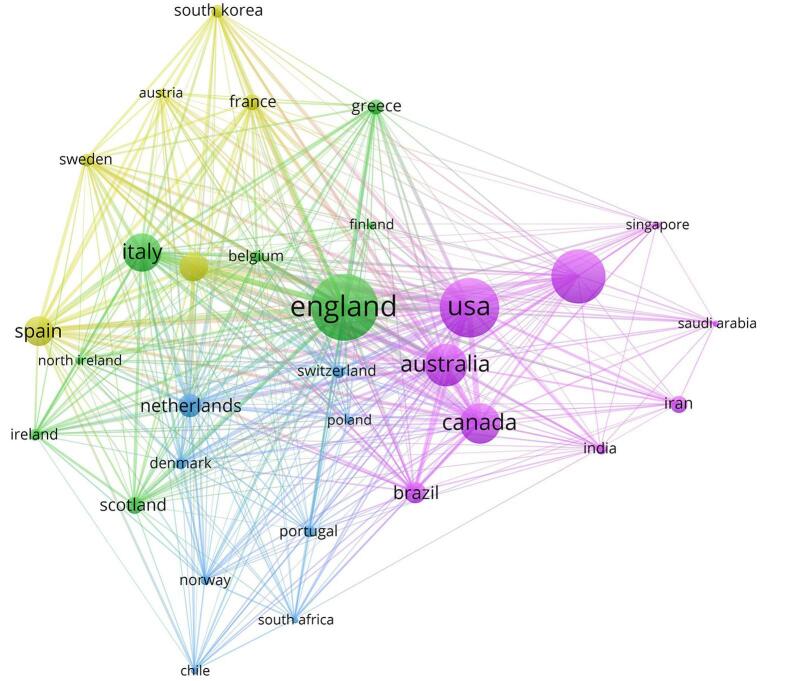
Distribution of publications among various countries (note the thick lines between England and the USA, England and Australia, England and Italy). Different colors show the various clusters.

**Figure 7.**
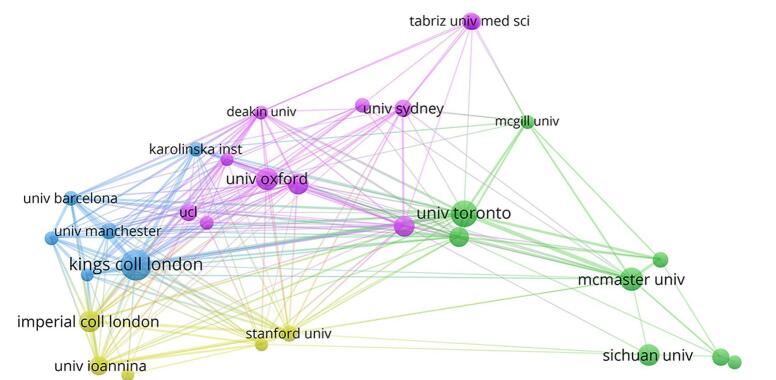
The distribution of publications among different organizations (note the thick lines between the University of Toronto and McMaster University, King’s College London and the University of Barcelona, and Imperial College London and Ioannina University). Different colors show the various clusters.

Our analysis revealed that in total, 4223 institutions have collaborated in this field. Among them, 41 have published more than 30 articles. King’s College London had the highest number of publications, followed by the University of Toronto and McMaster University. There were strong collaborations between the University of Toronto and McMaster University, King’s College London and the University of Barcelona, and Imperial College London and Ioannina University. Table 2 presents the top 10 institutions along with their number of publications in this field. Figs. 5 and 6 indicate that research on umbrella reviews by nations and organizations could have been key in the field. Each node represents a country or institution, with the node’s size reflecting the volume of published articles. The connections between nodes symbolize cooperation, with thicker links indicating stronger cooperative ties (Fig. 7).

**Table 2:** Top 10 leading institutions in the field of umbrella reviews

Number	Institution	Number of publications
1	King’s College London	95
2	University of Toronto	81
3	McMaster University	66
4	University of Oxford	65
5	Imperial College London	61
6	Sichuan University	61
7	University of Melbourne	58
8	Monash University	58
9	University of Ottawa	56
10	University of Ioannina	51

### Authors analysis

It has been found that a total of 14 598 authors have contributed to the field of umbrella reviews. Among them, 40 authors published more than 10 umbrella reviews each. Marco Solmi was noted to be the most productive author in terms of published papers, followed by John P. A. Ioannidis and Vali Musazadeh (Table 3).

**Table 3:** Top 10 authors and co-cited authors

Number	Author with high number of publications	Number of publications	Author with high number of citations	Number of citations	The most co-cited authors	Number of citations
1	Marco Solmi	37	John P. A. Ioannidis	3925	B. J. Shea	2055
2	John P. A. Ioannidis	27	Ioanna Tzoulaki	2871	David Moher	1067
3	Vali Musazadeh	25	Evangelos Kanoulas	2272	John P. A. Ioannidis	787
4	Paolo Fusar-Poli	24	Konstantinos K Tsilidis	1582	Edoardo Aromataris	698
5	Lee Smith	24	Lazaros Belbasis	1555	Julian Higgins	646
6	Nicola Veronese	24	Peter T. Katzmarzyk	1477	Gordon Guyatt	566
7	Andre F Carvalho	22	Kenneth E. Powell	1477	M. J. Page	428
8	Brendon Stubbs	21	Marco Solmi	1445	Dawid Pieper	406
9	Jae Il Shin	20	Paolo Fusar-Poli	1420	Valerie Smith	302
10	Jacopo Demurtas	17	David M. Buchner	1315	Michelle pollock	280

In terms of citations, John P. A. Ioannidis was the most cited author. Ioanna Tzoulaki and Evangelos Kanoulas followed closely (Table 3).

Furthermore, a co-authorship analysis revealed a strong collaborative network among authors in this field. Key collaborators, BJ Shea, David Moher, and John P. A. Ioannidis have significantly contributed to advancements in this domain (Table 3).

### Citation analysis

Among the included studies, 217 articles were cited more than 100 times. The most cited article was a study by Bero *et al*, published in the *BMJ journal* in 1998^[36]^. This study addressed the issue of translating research findings into routine clinical practice, highlighting the gap between clinical research and its implementation. The authors emphasized the importance of various interventions aimed at promoting behavioral change among healthcare professionals to facilitate the adoption of research findings. They acknowledged the challenge of distinguishing the effects of these interventions from contextual influences in individual trials. However, they argued that systematic reviews of high-quality studies offered the best evidence regarding the effectiveness of strategies for promoting behavioral change. The paper aimed to analyze systematic reviews of different dissemination and implementation strategies to identify evidence of their effectiveness and to evaluate the quality of these systematic reviews. In essence, the study focused on synthesizing existing evidence to understand how best to bridge the gap between research and clinical practice in healthcare settings (Table 4).

**Table 4:** Top 10 cited papers and 180-day usage count papers

Top 10 cited papers					Top 10 180-day usage count papers				
Title	First author	Year of publication	DOI	Number of citations	Title	First author	Year of publication	DOI	180-day usage count
Closing the gap between research and practice: an overview of systematic reviews of interventions to promote the implementation of research findings	Bero	1998	10.1136/bmj.317.7156.465	1628	Mobile Text Messaging for Health: A Systematic Review of Reviews	Hall	2015	10.1146/annurev-publhealth-031914-122 855	110
The Impact of eHealth on the quality and safety of health care: a systematic overview	Black	2011	10.1371/journal.pmed.1000387	785	Effectiveness of physical activity interventions for improving depression, anxiety, and distress: an overview of systematic reviews	Singh	2023	10.1136/bjsports-2022-106 195	79
Systematic review of reviews of intervention components associated with increased effectiveness in dietary and physical activity interventions	Greaves	2011	10.1186/1471-2458-11-119	729	Urban natural environments as nature-based solutions for improved public health—A systematic review of reviews	Bosch	2017	10.1016/j.envres.2017.05.040	74
Methodology in conducting a systematic review of systematic reviews of healthcare interventions	Smith	2011	10.1186/1471-2288-11-15	686	Social media use and well-being: What we know and what we need to know	Valkenburg	2022	10.1016/j.copsyc.2021.12.006	70
Changing provider behavior: an overview of systematic reviews of interventions	Grimshaw	2001	—	680	Exploring the impact of industrial robots on firm innovation under a circular economy umbrella: a human capital perspective	Luo	2023	10.1108/MD-02-2023-0285	60
Vitamin D and multiple health outcomes: umbrella review of systematic reviews and meta-analyses of observational studies and randomized trials	Theodoratou	2014	10.1136/bmj.g2035	654	The serotonin theory of depression: a systematic umbrella review of the evidence	Moncrieff	2023	10.1038/s41380-022-01661-0	54
The impact of child sexual abuse on health: a systematic review of reviews	Maniglio	2009	10.1016/j.cpr.2009.08.003	575	Dietary sugar consumption and health: umbrella review	Huang	2023	10.1136/bmj-2022-071609	51
Risks associated with obesity in pregnancy, for the mother and baby: a systematic review of reviews	Marchi	2015	10.1111/obr.12288	535	Teachers’ role in digitalizing education: an umbrella review	Wohlfart	2023	10.1007/s11423-022-10 166-0	51
Mediterranean diet and multiple health outcomes: an umbrella review of meta-analyses of observational studies and randomized trials	Dinu	2018	10.1038/ejcn.2017.58	528	The efficacy of psychotherapies and pharmacotherapies for mental disorders in adults: an umbrella review and meta-analytic evaluation of recent meta-analyses	Leichsenring	2022	10.1002/wps.20941	48
The impact of mHealth interventions: systematic review of systematic reviews	Marcolino	2018	10.2196/mhealth.8873	524	Effects of screentime on the health and well-being of children and adolescents: a systematic review of reviews	Stiglic	2019	10.1136/bmjopen-2018-023191	48

The second highly cited paper was a study by Black *et al*, published in the *PLOS Medicine* journal in 2011^[37]^. The study aimed to conduct a systematic review of systematic reviews to assess the effectiveness and consequences of various eHealth technologies on the quality and safety of healthcare. The study found that while there is considerable international interest and investment in implementing transformative eHealth technologies, the empirical evidence supporting their benefits is limited. Despite policymakers’ support, there was insufficient empirical evidence to substantiate many claims regarding the effectiveness and safety of eHealth interventions. The study identified a gap between the perceived and demonstrated benefits of eHealth technologies, highlighting the need for robust research on their efficacy, risks, and cost-effectiveness (Table 4).

The third most cited paper was a study by Greaves *et al*, published in BMC Public Health in 2011^[38]^. This systematic review of reviews aimed to identify intervention components associated with increased effectiveness in promoting dietary and/or physical activity changes among individuals at risk of type 2 diabetes. Through the analysis of 30 included reviews comprising 129 analyses, the study found that interventions targeting both diet and physical activity, incorporating well-defined behavior change techniques, and engaging social support were associated with increased effectiveness. Additionally, interventions with higher contact frequency and utilizing a cluster of “self-regulatory” behavior change techniques, such as goal-setting and self-monitoring, were linked to greater effectiveness. The study highlighted the importance of considering these specific components to maximize the efficiency of programs for diabetes prevention (Table 4).

The number of times the article was utilized in 180 days indicated how many times a user’s information demands were satisfied, as evidenced by links that took the user to the publisher’s website to read the complete article or by saving the metadata for later use. It took time for large usage numbers to result in high citation counts. Nevertheless, they offered the benefit of novelty, and scholars frequently draw from more recent works. Furthermore, a secondary increase in the literature’s future use was facilitated by earlier works with larger citation counts^[39]^. To some extent, the number of 180-day usages can represent the frontiers and hotspots of current research^[40]^.

The study by Hall *et al* published in the *Journal of Annual Review Public Health* in 2015 had the highest 180-day usage count^[41]^. The objective of this systematic review of reviews was to identify mobile text-messaging interventions aimed at enhancing health and altering behavior, to derive practical recommendations. Through the compilation and assessment of existing systematic research reviews and meta-analyses, the researchers aimed to organize and summarize the evidence base for text-messaging interventions, extract best-practice recommendations from multiple reviews, and examine implications for future research. The review revealed that most text-messaging interventions, targeting diabetes self-management, weight loss, physical activity, smoking cessation, and medication adherence for antiretroviral therapy, demonstrated effectiveness. However, there was limited evidence available across studies and reviews to guide recommended intervention characteristics (Table 4).

A study by Singh *et al* that was published in the British Journal of Sports Medicine in 2023 was the second study with the greatest 180-day usage count^[42]^. The goal of this umbrella review was to consolidate findings regarding the impact of physical activity on symptoms of depression, anxiety, and psychological distress among adults. The analysis encompassed 97 reviews, which collectively incorporated data from 1039 trials and 128 119 participants. Various demographic groups were represented, including healthy adults, individuals with mental health conditions, and those with chronic illnesses. The results indicated that physical activity yielded moderate benefits in alleviating depression, anxiety, and psychological distress compared to standard care across all studied populations. Particularly noteworthy benefits were observed in individuals with depression, HIV, kidney disease, pregnant and postpartum women, as well as healthy individuals. Notably, engaging in higher-intensity physical activities was linked to more pronounced symptom improvements, while the efficacy of interventions tended to diminish with prolonged durations (Table 4).

The third study regarding the 180-day usage count was conducted by Bosch *et al* in the *Environmental Research Journal* in 201^[43]^. The purpose of this paper was to evaluate the evidence on the positive effects of exposure to natural surroundings on public health and investigate how this understanding may be incorporated into the nature-based solutions paradigm for building sustainable and healthy communities. The study investigated relationships between public health and natural environments through a systematic review of reviews, taking into account specific health outcomes like cardiovascular mortality as well as socio-behavioral/cultural ecosystem services (like stress reduction and physical activity) and regulating ecosystem services (like heat reduction). The results showed compelling evidence for enhanced effect and reduced heat from urban natural surroundings, which may have an impact on mortality from cardiovascular disease^[43]^ (Table 4).

### Cluster citation analysis

**Figure 8.**
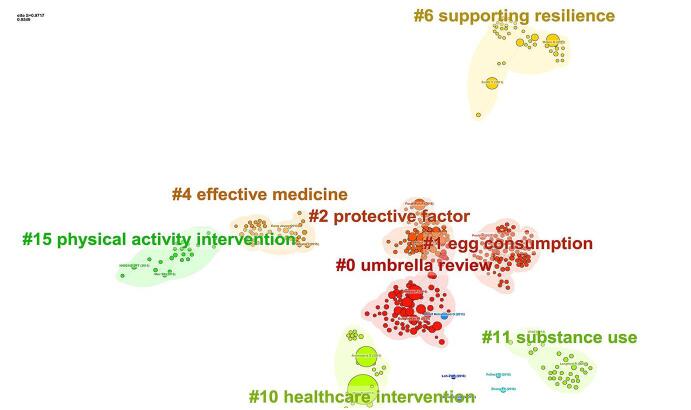
Cluster analysis-based topics.

The study identified a total of 207 distinct clusters derived from the thematic analysis of article topics. Among these, eight clusters emerged as notably significant (Fig. 8). These encompassed a wide spectrum of themes, including “Umbrella Review,” “Egg Consumption,” “Protective Factors,” “Effective Medicine,” “Physical Activity Intervention,” “Supporting Resilience,” “Substance Use” and “Healthcare Intervention.”

**Figure 9.**
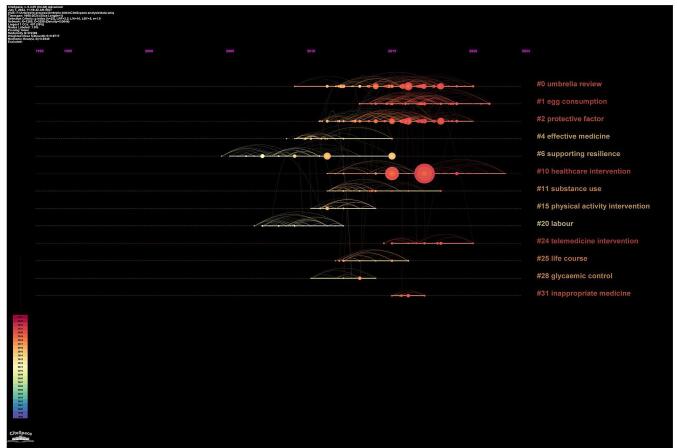
Time trend analysis of the topic clusters.

A chronological examination of these clusters through timeline analysis illuminated the evolution of research focus within this domain. It was discerned that the exploration commenced with the thematic cluster centered around “Supporting Resilience” gradually transitioning towards the more contemporary emphasis on “Healthcare Intervention” (Fig. 9).

### Keyword analysis

**Figure 10.**
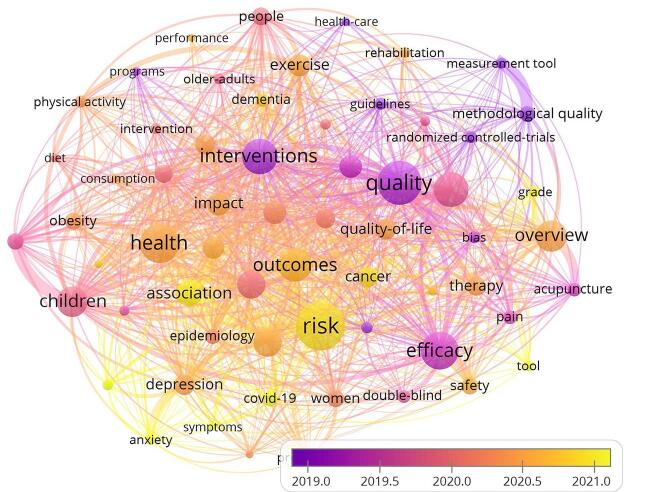
Overlay visualization of the co-occurrence keywords.

A total of 1501 keywords were identified through the keyword analysis. Fig 10 presents an overlay visualization of the keywords. Recently, keywords such as COVID-19, mental health, symptoms, and anxiety have gained popularity. On the other hand, older keywords included measurement tools, methodological quality, randomized controlled trials, guidelines, health care, and interventions.

## Discussion

Systematic reviews are crucial components of evidence-based medicine, ranking high in the evidence hierarchy^[21]^. A well-executed systematic review can effectively keep healthcare providers updated with the latest evidence-based practices^[44]^. These reviews used a rigorous methodological approach to help decision-makers identify the best available evidence^[22,45]^. Over the past 20 years, the number of systematic reviews published across various fields has grown exponentially. However, this increase was not entirely beneficial^[46]^. When multiple systematic reviews and meta-analyses on the same topic are released within the same year, their results can be inconclusive, causing uncertainty for decision-makers. Therefore, it is essential for decision-makers to critically evaluate the quality and confidence of evidence presented in systematic reviews. Due to the need for a comprehensive synthesis, an additional step of consolidating existing systematic reviews has been established^[45]^.

The field of umbrella reviews has witnessed ascending growth, particularly in recent years. Our analysis indicated a significant increase in publications, especially post-2019, with over 700 articles published in 2023 alone. This trend aligned with Hossain *et al* findings, which noted a marked rise from 2015 onward^[28]^. This increase can be attributed to the urgent need for high-level evidence synthesis during global health crises such as COVID-19, emphasizing the role of umbrella reviews in rapidly consolidating diverse research findings.

Umbrella reviews, which are systematic reviews of existing reviews, offer a comprehensive assessment of information on a specific topic. They reach clear conclusions by systematically reviewing variables, thus integrating findings from previously published systematic reviews or meta-analyses. Umbrella reviews are useful for quickly assessing a large body of evidence and comparing results from prior reviews. Typically, they impose overall coherence by categorizing broad issues into specific populations, interventions, or both^[20]^.

Consistent with prior studies, high-income countries like the USA, UK, and Canada dominated the landscape of umbrella reviews. However, our study highlighted the emergence of China as a significant contributor, reflecting a more globalized research effort since 2019^[35]^. Institutions such as King’s College London and the University of Toronto continue to lead, fostering strong international collaborations. Despite this, the underrepresentation of low- and middle-income countries (LMICs) remained a concern, as previously noted by Hossain *et al*^[28]^. This gap highlights the need for capacity-building initiatives to ensure more genuine contributions and benefits from evidence synthesis.

Umbrella reviews can be considered a method for tertiary-level evidence synthesis. The increasing recognition of its crucial role in integrating information is seen in the notable rise in the number of published umbrella reviews during the past years, particularly between 2021 and 2023. It provided a comprehensive analysis of several sources of information, enabling healthcare practitioners to make well-informed choices about patient treatment based on the highest quality evidence^[4,47–49]^. While a single meta-analysis aggregated results for studies of a single treatment, an umbrella review has the potential to be even more effective by comparing different treatments from multiple existing meta-analyses. This method thoroughly compared evidence across different treatments as opposed to just one treatment, aiming to derive a higher-level meaning and ultimately issue a call to action. This can strengthen the efforts of holistic and preventative medicine, such as working to help patients become self-motivated to make lifestyle changes.

The findings of our study emphasized the growing importance of umbrella reviews in evidence-based medicine, aligning with the observations of Choi and Kang^[4]^. Umbrella reviews provide a high-level synthesis of systematic reviews and meta-analyses, offering clinicians and decision-makers a comprehensive view of healthcare topics. This capability is particularly valuable in clinical settings, where timely and well-informed decisions are critical.

Our analysis demonstrated a significant increase in the number of umbrella reviews, particularly in the last decade. This trend mirrored the broader increase in scientific literature, underscoring the utility of umbrella reviews in managing large volumes of data. However, challenges persist, especially in determining the certainty of evidence and ensuring methodological consistency, as noted by Choi and Kang and Asgarizadeh *et al*^[4,50]^. These challenges highlighted the need for standardized guidelines to enhance the reliability and interpretability of umbrella review findings.

In surgical research, umbrella reviews remain underutilized despite their potential to synthesize practical, evidence-based knowledge. A recent study argued that the effectiveness of URs in this field depends heavily on the quality and bias limitations of the included systematic reviews^[51]^. Our findings supported this assertion, suggesting that as methodological standards for URs improve, their adoption in surgical research could yield more insights.

Despite the recent interest in umbrella reviews as a research methodology, only 37 journals, including *BMJ Open, Plos One*, and *Systemic Reviews*, showed substantial contributions. Access to these platforms enabled researchers to make educated decisions, formulate guidelines, and identify areas where further study was needed. Co-citation findings highlighted the multidisciplinary character of scientific research, as indicated by the citation patterns across fields. Notably, the dual-map overlay emphasized the influence of evidence in several disciplines on one another, underscoring the value of multidisciplinary collaboration in developing knowledge and understanding in the field of umbrella meta-analysis technique.

Given the rapid growth of the research landscape as indicated in our analysis, the role of URs in synthesizing and distilling scientific evidence was expected to expand. By providing a structured overview of diverse studies, URs can help bridge gaps between research and practice, ultimately supporting more informed and efficient healthcare decision-making^[4]^. Due to the efforts required to conduct an umbrella review, it is evident why only a small group of institutions and journals published the vast majority of umbrella reviews. While consolidation of publishing origin may be good for homogeneity in comparing reviews, critique must be applied to the models and reasoning to ensure that the conclusions are applicable in a healthcare field with diverse demographics and context-specific treatment options.

The current study aligned with the findings of prior umbrella reviews, demonstrating the utility of evidence synthesis in addressing diverse healthcare challenges. Conn and Coon Sells emphasized the effectiveness of physical activity interventions in minority populations, although with variability in outcomes^[52]^. Our analysis similarly identified physical activity as a recurring focus in umbrella reviews, underscoring its critical role in promoting health equity. This convergence highlighted the growing research interest in tailored interventions to address disparities across populations.

Khalil *et al* explored interventions for enhancing medication safety in acute care, such as educational programs and systemic modifications^[53]^. Their findings highlight the heterogeneity of intervention outcomes. In parallel, our bibliometric analysis revealed that umbrella reviews often deal with methodological challenges, particularly when synthesizing evidence from heterogeneous sources. This reinforced the necessity of advanced synthesis methods to improve consistency and reliability in fields like medication safety.

The analysis of countries and institutions with a prominent contribution from umbrella reviews sheds light on the global landscape of engagement. While countries like England, the USA, and China are prominently featured, low- and middle-income countries are notably less common on the list. This discrepancy underscored existing disparities in research infrastructure and capacity, with implications for the need for equitable adoption of evidence-based practices worldwide. Therefore, efforts to promote capacity building and facilitate international collaboration should prioritize the participation of low- and middle-income countries to enhance the diversity and representativeness of umbrella meta-analysis research.

Although Sufrate-Sorzano *et al* sought to identify interventions for suicidal behaviors^[54]^. Our study highlighted the increasing emphasis on mental health in recent umbrella reviews. The identified trends suggested that mental health remains a critical area, warranting comprehensive evidence synthesis to inform policy and practice. This aligned with the broader thematic evolution observed in our study, wherein emerging healthcare concerns such as mental health and behavioral interventions have gained prominence.

Given the purpose of our bibliometric analysis, the prominence of the “Umbrella review” cluster, as shown in Fig. 8, was an expected result. Since analyzing umbrella reviews was the main goal of this study, it made sense that the term “Umbrella review” would rank as the most commonly appearing keyword. The magnitude and importance of this cluster demonstrated the important role umbrella reviews played in integrating research findings from many fields.Apart from the “Umbrella review” cluster, Fig. 7 presents some additional notable clusters that corresponded to different study areas. Within the larger field of medical study, clusters like “#4 effective medicine” “#2 protective factor” and “#10 healthcare intervention” highlighted areas of attention.

Timeline cluster analysis demonstrated a progressive shift in research emphasis within the field. The research started with a theme cluster focused on “Supporting Resilience” demonstrating an initial curiosity in comprehending the elements that contributed to resilience in healthcare settings. Over time, there was a clear movement towards focusing more on “Healthcare Intervention” which involved examining the effectiveness and implementation of interventions in clinical practice. The changing nature of research in the umbrella meta-analysis approach is emphasized by this temporal history, reflecting the changing research priorities to tackle new healthcare concerns.

The relationship between existing and new domains in healthcare research and practice was revealed by the keyword analysis, demonstrating the changing character of this field. The newer challenges and fields of interest in healthcare were seen in the rise of terminology such as COVID-19, mental health, symptoms, and anxiety. Conversely, terminology like measuring instruments, methodological quality, and randomized controlled trials, which were widely accepted, highlighted the core principles and enduring practices in the field. It played a significant role in synthesizing evidence and ultimately informing healthcare treatments and interventions. The most-cited umbrella review highlighted this strength by comparing different strategies for implementing medical findings into practice.

However, among the extensive range of keywords, there was probably a category of infrequent phrases that signify specialized or unexplored areas. Through the process of evaluating and detecting these infrequent terms, researchers can determine certain regions that require further compilation of existing evidence. Focusing on these less-explored fields might aid in broadening the knowledge base to serve more healthcare needs. Thus, encouraging new studies in these less frequent fields might improve umbrella meta-analysis and its relevance to varied healthcare concerns.

## Limitations

Our study benefited significantly from the extensive data collection facilitated by the Web of Science Core Collection. However, some limitations must be acknowledged. First, the scope of our search was confined to a single database, potentially leading to the exclusion of relevant research available from other sources. Additionally, while our analysis primarily relied on objective tools such as VOSviewer and CiteSpace, it is equally essential to account for subjective factors crucial to evidence-based medicine, such as ethical considerations and patient perspectives.

Moreover, our focus on published umbrella reviews may have inadvertently excluded other forms of meta-reviews not explicitly categorized under this term. Integrating the findings of umbrella reviews with smaller-scale empirical studies is critical for informed decision-making in clinical settings. Given that umbrella reviews represent the highest level of evidence synthesis, their conclusions significantly influence healthcare guidelines. Therefore, the methods and conclusions of such reviews require scrutiny to ensure their robustness and relevance.

Despite these limitations, our study underscored the importance of refining content review methodologies to achieve a more comprehensive approach to evidence synthesis. Furthermore, there was a pressing need for healthcare professionals including physicians, nurses, and allied health practitioners to deepen their understanding of umbrella meta-analysis as a research methodology. By actively engaging in such research, they can bridge the gap between academic inquiry and clinical application, ultimately enhancing patient care and advancing the overall quality of healthcare services.

## Conclusion

Umbrella reviews have been at the forefront of enhancing patient outcomes. By comparing meta-analyses in a single systematic review, umbrella reviews do not just aggregate evidence; they transform it into practical guidelines. These comprehensive reviews provide valuable insights that can be used by all healthcare professionals, including physicians, nurses, residents, allied health workers, and policymakers, to make informed decisions and suggest novel lifestyle or preventative interventions that lower-level studies might not reveal. Our study highlighted the rapid growth and significant impact of umbrella reviews and provided insights into the current trends, key contributors, and geographical distribution of this research. This analysis demonstrated how umbrella reviews can bridge gaps in existing research, guide clinical practices, and shape future healthcare policies.

Moving forward, it is crucial to critique and refine the standards and methods of umbrella reviews to understand their limitations and enhance their future application. When implemented effectively, umbrella reviews empower all healthcare professionals and policymakers to quickly integrate higher-level evidence into practice, ultimately improving the quality of healthcare for patients everywhere. By fostering an inclusive approach to research and evidence synthesis, umbrella reviews play a pivotal role in advancing evidence-based medicine and improving patient care across various healthcare settings.

## Data Availability

Data from the study can be provided by the corresponding author upon reasonable request.

## Research registration unique identifying number (UIN)

The current study is a bibliometric study and UIN is not applicable for this study.
